# More than causing (epi)genomic instability: emerging physiological implications of transposable element modulation

**DOI:** 10.1186/s12929-021-00754-2

**Published:** 2021-08-07

**Authors:** Pu-Sheng Hsu, Shu-Han Yu, Yi-Tzang Tsai, Jen-Yun Chang, Li-Kuang Tsai, Chih-Hung Ye, Ning-Yu Song, Lih-Chiao Yau, Shau-Ping Lin

**Affiliations:** 1 Institute of Biotechnology, College of Bio-Resources and Agriculture, National Taiwan University, Taipei, Taiwan; 2 Department of Biomedical Engineering, Stony Brook University, Stony Brook, NY USA; 3 Department of Biomedical Engineering, National Taiwan University, Taipei, Taiwan; 4 Agricultural Biotechnology Research Center, Academia Sinica, Taipei, Taiwan; 5 Center of Systems Biology, National Taiwan University, Taipei, Taiwan; 6 The Research Center of Developmental Biology and Regenerative Medicine, National Taiwan University, Taipei, Taiwan

**Keywords:** Transposable elements (TEs), Functional RNAs, Epigenetics, Enhancers, Differentiation, Evolution

## Abstract

Transposable elements (TEs) initially attracted attention because they comprise a major portion of the genomic sequences in plants and animals. TEs may jump around the genome and disrupt both coding genes as well as regulatory sequences to cause disease. Host cells have therefore evolved various epigenetic and functional RNA-mediated mechanisms to mitigate the disruption of genomic integrity by TEs. TE associated sequences therefore acquire the tendencies of attracting various epigenetic modifiers to induce epigenetic alterations that may spread to the neighboring genes. In addition to posting threats for (epi)genome integrity, emerging evidence suggested the physiological importance of endogenous TEs either as cis-acting control elements for controlling gene regulation or as TE-containing functional transcripts that modulate the transcriptome of the host cells. Recent advances in long-reads sequence analysis technologies, bioinformatics and genetic editing tools have enabled the profiling, precise annotation and functional characterization of TEs despite their challenging repetitive nature. The importance of specific TEs in preimplantation embryonic development, germ cell differentiation and meiosis, cell fate determination and in driving species specific differences in mammals will be discussed.

## Introduction

Transposable elements (TEs) were first discovered in maize in the late 1940s [1]. TEs were considered endogenous “junk sequences” or “selfish genomic sequences”. This is mainly due to their virus-based genomic features that are designed to amplify themselves or move around the host genome at the cost of genomic instability of the host cells. In mammals, TEs comprise roughly 45% of the genomic sequences [2]. TEs have been classified into two categories: DNA transposons and retrotransposons. DNA transposons such as tc1/mariner cut and paste themselves to reach transposition. Retrotransposons copy and paste via an RNA intermediate followed by reverse transcription to achieve retrotransposition. Retrotransposons are further classified into long terminal repeats (LTRs), such as endogenous retroviruses (ERVs), and non-LTRs, such as long interspersed nuclear elements (LINEs) and short interspersed nuclear elements (SINEs). Long since their invasion into an ancient host genome, evolutionarily older TEs have been mutated or truncated and eventually lost their ability to transpose within the genome [3]. On the other hand, evolutionarily younger retrotransposons are the most dangerous threats to genome integrity since they maintain the capability of transposition [4]. The occurrence of TE insertions can cause genomic instability [5–7] and transcriptional deregulation. Under pressure from TE-derived hazards, both transcriptional and posttranscriptional defense systems evolve in the host [8]. Although many TEs are neutralized in the host, more than 120 disease-causing TE insertions have been documented in humans [9].

Most TEs contain numerous copies, which makes it difficult for scientists to map each TE sequences to the exact genomic location with second-generation short read sequencing platforms. However, in the last few years, advanced third-generation sequencing technologies (reviewed by Amarasinghe, 2020 [10]) have enabled the detection of long sequencing reads and the identification of location-specific small variations in each TE family member. With the associated optimized bioinformatics packages, getting the precise localization of TEs is now practical. The improvement of technologies helps scientists reveal that these mutated TEs are susceptible to substantial epigenetic modulation, which also spreads to adjacent genomic regions and therefore affects the expression of neighboring genes during the development and physiological function of organisms [11]. TEs actually function as a double-edged sword in host cells. In this review, we introduce not only how TEs are regulated by host organisms and what happens when they are dysregulated but also recent evidence showing the physiological properties of TEs.

## The threats associated with TEs in host cells

TEs are usually silenced by epigenetic modifications, including DNA methylation and histone modifications [12–14]. Some TEs are packaged into heterochromatin structure associated with nuclear lamins [15]. However, aging-associated or other aberrant micro- or macroenvironment-induced epigenomic defects may cause dysregulation of TEs. These TE deregulations may induce genomic instability [16, 17] and diseases, including neurodevelopmental disorders (reviewed by Lapp, 2019 [18]), neurodegeneration [19, 20], autoimmunity [21] and cancer [22].

Some of the activated TEs have the ability to insert into other genomic sites, the processing of which can cause DNA double-strand breaks (DSBs). Gasior et al. documented that the transfection of LINE-1 into HeLa cells induces DSBs and G2/M cell cycle arrest [23]. Although DNA repair system can fix most of the TE transposition-induced DSBs, this process still disrupts genome stability and may cause chromosomal rearrangement, gene mutation or alternative splicing. For example, LINE-1 insertion usually creates a large genetic deletion [24] and may lead to chromosomal rearrangement [25]. Moreover, scientists have found that more than eighty percent of Alu elements inserted into the exons of mRNAs cause a frameshift or a premature termination codon that affects the expression of those coding genes [26]. In addition, TEs inserted into introns also cause problems; for example, Alu and LINE-1 induce alternative splicing to affect transcript integrity [27, 28]. If TEs insert into DNA repair genes such as breast cancer type 2 susceptibility protein (*BRCA2*) [29] or tumor suppressor genes such as adenomatous polyposis coli protein (*APC*) [30] and retinoblastoma protein 1 (*RB1*) [31], they may cause genome instability or tumor formation, respectively. Furthermore, several cancers, including lung cancer, renal cancer, breast cancer, etc*.*, are correlated with DNA hypomethylation and TE deregulation [5, 7, 32, 33]. Kong et al. also observed similar phenomena by analyzing the transcriptomes of over twenty different cancers from the Cancer Genome Atlas database [34]. Lee et al. showed the consequences of TE insertion in different cancer samples by performing whole genome sequencing. They observed that LINE-1-inserted genes are usually dysregulated and associated with cancer formation [35].

**Fig. 1:**
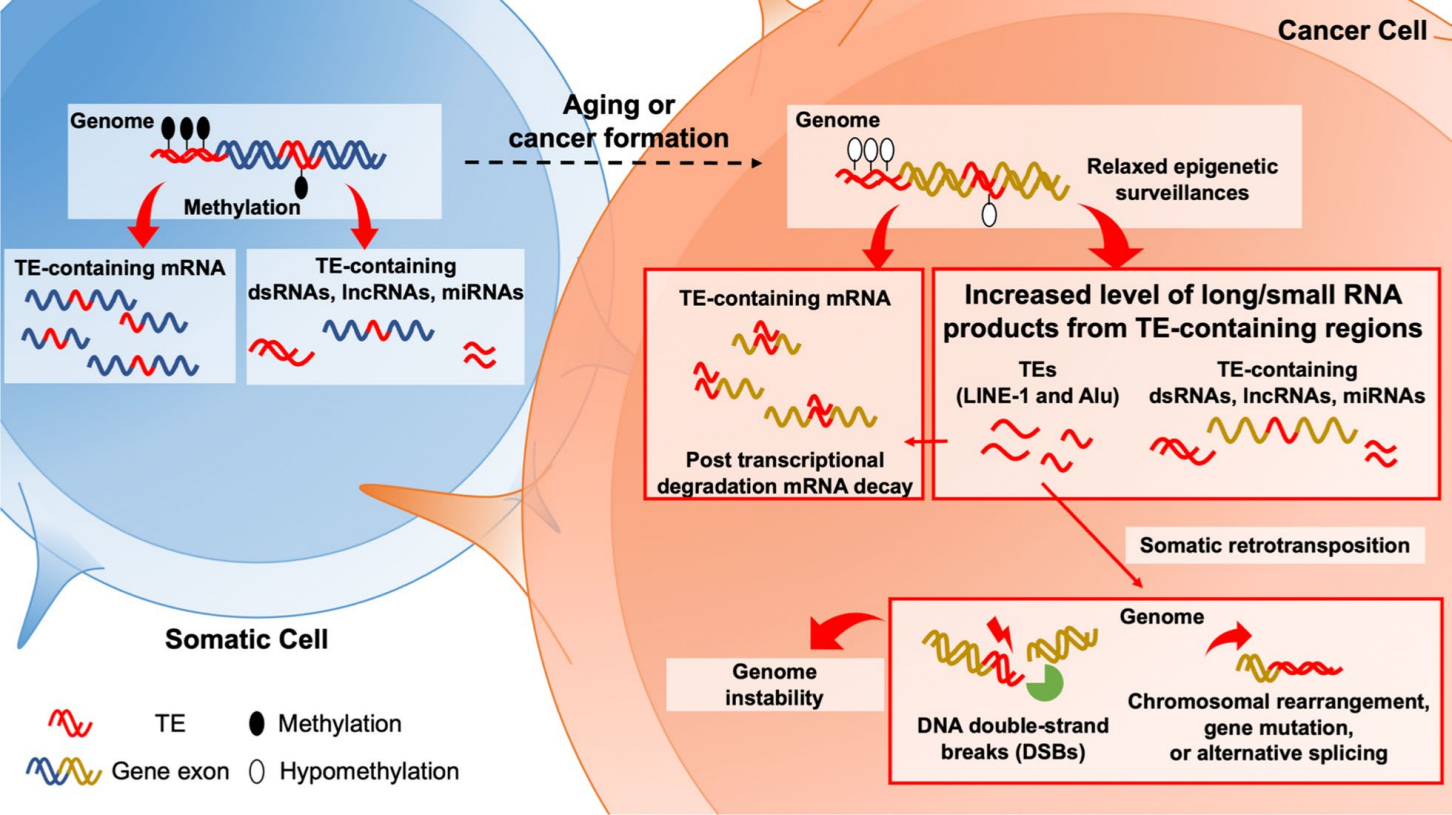
Global DNA hypomethylation leads to TE reactivation in cancer cells. In somatic cells, TEs are mostly silenced by epigenetic modifications, such as DNA methylation. However, a detectable portion of transcripts expressed from normal somatic cells (blue cells in the left panel) are still characterized as TE-containing transcripts with potential physiological functions. In cancer cells (right panel), global hypomethylation is observed, including removal of the repressive marks on TEs. Consequently, increased levels of long/small RNA products from TE-containing regions are observed. Excessive sense or antisense TE transcripts might cause the targeted degradation of TE-containing mRNAs based on reverse complementarity. In addition, TE transcripts may cause alternative splicing, gene mutation and genomic instability, including DNA DSBs and chromosomal rearrangement via somatic retrotransposition. Modified from [9]

Even without transposition, the presence of TE sequences may still disrupt (epi)genome stability. TE enriched sequences can be found in the flanking sequences of DSBs in cancer cells. It is suggested that during DNA replication, short inverted repeats such as the Alu element may form a secondary hairpin structure, which can lead to replication stalling and even DNA DSB formation [36, 37]. On the other hand, global hypomethylation of endogenous TEs in cancer cells might further induce alternative promoter activation. For example, Jang et al*.* investigated 15 cancer types in 7,769 tumor and 625 normal tissue datasets to identify 129 TE-related promoter activation events. The authors showed that the global profile of these TEs is associated with 106 oncogenes across 3,864 tumors, and TEs are the cause of oncogenic activation [38, 39], which may further contribute to tumor initiation and progression [7, 22, 38]. As shown in Fig. 1, DNA damage, chromosomal rearrangement [35, 40–43], alternative splicing and gene expression are induced by abnormal TE activation and, in some cases, subsequent insertion in cancer cells. These TE insertions were shown to further activate aberrant and recombinant gene transcription [44].

Global DNA hypomethylation associated with de-repression of TEs can be observed in normal aging cells as well [45], but may be reversible. As shown in our recent study, transient ectopic expression of an epigenetic cofactor, DNMTT3L, in aging fibroblasts is sufficient to inhibit senescence progression and facilitate epigenetic repression of some aging associated derepressed genes and TEs [46]. It is therefore possible to develop strategies in mitigating aging-associated defects via increasing epigenomic surveillance.

## Modulating TEs in host cells

Several strategies for protecting host cells from TE-derived disruption have emerged during evolution. Both transcriptional and posttranscriptional pathways are involved in TE regulation. The consummate management of TEs is presumably performed with the coordination of DNA methylation, histone modifications, and small RNA-mediated RNA degradation in mammalian cells.

### DNA methylation

Among the TE modulation machineries, DNA methylation is used for long-term TE surveillance in mammals. Both LTR and non-LTR retrotransposons are inhibited by DNA methylation [13, 47]. In DNA methylation-deficient models, the expression of TEs was increased significantly and caused developmental defects [13, 47, 48].

Thirty years ago, Prof. Timothy Bestor hypothesized that the DNA methylation machinery evolved from immune mechanisms in prokaryotes designed to protect against phage infection into gene expression and genome structural modulation in large-genome plants and vertebrate animals, including silencing of TEs and other repeat sequences to reduce their exposure to the transcriptional machinery [60]. Recently, Zhou et al*.* examined the complete DNA sequences of 53 organisms, and the results supported the original hypotheses that DNA methylation enables TE-driven genome expansion. The results of these analysis also indicated that DNA methylation spreads to the flanking host DNA sequences associated with the inserted TEs [59].

DNA methylation in mammals predominantly take place on the 5^th^ carbon of cytosine (5-methylcytosine; 5mC), catalyzed by a family of DNA methyltransferases (DNMTs) [49]. DNMT1 transfer the methyl group from S-adenyl methionine (SAM) mainly to hemimethylated DNA template during DNA replication to maintain DNA methylation mark from mother strand to daughter strand [50–52]. DNMT3A and DNMT3B target unmethylated DNA template to introduce de novo methylation mark during development [53, 54]. Another pluripotent stem cells and developing germ cells enriched DNNMT3 family member, DNMT3-Like (DNM3L), lacks functional catalytic domain but serve as an important co-factor to facilitate DNMT3A and DNMT3B for de novo methylation on TEs and beyond [55, 56]. DNA methylation not only results in the transcriptional repression of TEs but can also result in C-T deamination and inactivate those sequences permanently at a more advanced level [57–59].

### Histone modification

Post-translational histone modifications, including acetylation, methylation and ubiquitylation, collaborate with DNA methylation machineries to accomplish multiple layers of epigenetic modulation [61]. The repressive marks H3K9me2/3 and H3K27me3 are responsible for silencing different types of transposons at different differentiation stages and coordinating TE regulation with other silencing strategies [62]. H3K9me2/3 is linked to the maintenance of DNA methylation mediated by ubiquitin-like, containing PHD and RING finger domains protein 1 (UHRF1) in ESCs [63–65]. Furthermore, H3K9me2/3 may be connected to the role of UHRF1 in the maintenance of 5mC levels at intracisternal A-type particles (IAPs) in preimplantation embryos [63].

Krüppel-associated box zinc finger protein (KRAB-ZFP)- KRAB-associated protein-1 (KAP1)-mediated silencing is an interesting system that coevolves with TEs and is critical in early embryogenesis. Its targeting might be adapted to new retroelements through evolutionarily changing the DNA-binding region [8, 66]. KRAB-ZFPs are transcription factors that use the C-terminal ZFP to target TE sequences and the N-terminal KRAB domain to bind tripartite motif-containing 28 protein (TRIM28)/KAP1, which is a scaffold that recruits epigenetic modifiers. The H3K9-specific methylase SET domain bifurcated histone lysine methyltransferase 1 (SETDB1) is recruited to introduce repressive histone modifications, such as H3K9me3 [67, 68]. In addition, ZFP/KAP1 was reported to interact with DNMT3A/3B and to play a role in the maintenance of DNA methylation in embryonic stem cells (ESCs) [69].

### Small RNA-mediated TE regulation

The small RNA-mediated pathway is another mechanism that manages gene expression in a sequence-dependent manner. Additionally, it is vital for controlling and counteracting TE transcripts, especially at the developmental stages when repressive DNA methylation and histone marks are modified during epigenetic reprogramming. In germ cells and early embryos, the DNA methylation profile changes dynamically during development [70]. RNA interference (RNAi) was indicated to regulate the expression of the retrotransposons murine endogenous retrovirus-leukemia protein (MuERV-L) and IAPs in preimplantation mouse embryos [71]. P-element-induced wimpy testis protein (PIWI)-interacting RNA (piRNA), on the other hand, is the best studied small RNA-mediated epigenetic regulator that functions to repress mobile genetic elements in germ cells [72]. The PIWI-piRNA pathway is also highly conserved across the animal kingdom to mitigate the threat of retrotransposition in germ cells [73]. piRNAs are approximately 26–34 nt single-stranded RNAs with 3’-end-2’-O methylation that are processed from long single-stranded transcripts, including TE transcripts. The biogenesis of piRNAs requires PIWI proteins, a specific clade of Argonautes, and other piRNA biogenesis-associated proteins in the nuage/germ granules immediately outside the nuclear envelope of developing germ cells [74–76]. Located in germ granule cement, the PIWI-piRNA pathway is generally considered a posttranscriptional silencing mechanism. With guidance by piRNA, PIWI proteins target sequence-complementary transposon transcripts and destroy RNAs by cleavage. Cleavage degrades TE transcripts during piRNA biogenesis and therefore blocks the reverse transcription and retrotransposition of these TE elements. The “ping-pong cycle” of secondary piRNA biogenesis that involves targeting and cutting sense- and antisense-strand TEs via the PIWI-piRNA complex with mature antisense and sense TE sequence-derived piRNAs, respectively, is particularly efficient at minimizing the expression of full-length TE transcripts [56, 77]. In addition, the PIWI-piRNA complex enters the nucleus, targets nascent TE transcripts and recruits epigenetic silencers, including histone methyltransferases, and even de novo DNA methylation for the long-term maintenance of transcriptional silencing [78–80].

Joint protection by DNA methylation, histone modification, and small RNA-mediated regulation defends against invasion by TEs. The crosstalk between these strategies affords different layers of retroelement regulation and modulates the orchestration of gene expression.

## Can’t eliminate them, use them: the physiological functions of TEs in host cells

**A**ccumulating evidence suggest that TEs acquire important physiological functions through evolution. Several LTR retrotransposon-derived genes have been discovered in the human genome, domesticated to neogenes of functional proteins. These include sushi/Mart [81], paraneoplastic Ma antigens protein (PNMA), activity-regulated cytoskeleton-associated protein (ARC), skin-specific retroviral-like aspartic protease/aspartic peptidase retroviral like 1 protein (SASPase/ASPRV1) [82], SCAN (SRE-ZBP, CTfin-51, AW-1 and 2 Number 18 cDNA protein) family members [83], recombination activating 1 protein (RAG1) and recombination signal sequences (RSSs) [84]. Those TE containing sequences are important in a myriad of biological processes, such as stem cell properties, tissue development, inflammation, V(D)J recombination and neurophysiology [84–89]. In addition, through big data analysis, Kong et al. also found that TE expression is correlated with the regulation of cytokine responses and induces the infiltration of some types of immune cells in cancer [34].

The properties of TEs in attracting epigenetic modifiers also enable the inserted TEs to become functionally relevant genomic features. By performing comprehensive chromatin immunoprecipitation (ChIP)-seq analyses in human and mouse leukemia cell lines (K562 and MEL) and lymphoblast cell lines (GM12878 and CH12), researchers have shown that TE sequences are present in 20% of transcription factor binding sites in immune cells in which neighboring areas show open chromatin marks, including DNA hypomethylation, H3K4me1, H3K4me3 and H3K27ac. In addition, LTRs comprise the majority of TE-derived binding peaks in human [90]. Furthermore, full-length retrotransposons are composed of a complete transcription unit, including a strong promoter [91–93]. Newly inserted or endogenously derepressed TEs may drive the transcription of neighboring genes or intergenic regions and evolve into functional RNAs. On the other hand, genome-wide chromatin profiling data and high-throughput sequencing have revealed the expression and lineage-specific distributions of multiple TE subfamilies [91, 94, 95]. Some enhancers are believed to have evolved from TEs [96]. TE-associated enhancers are involved in many developmental processes [97–99]. The functions of transposable elements in mammals are separated into two major categories: TE-containing functional RNAs and TE-containing *cis-*regulatory elements.

### TE-containing transcripts functioning in trans

#### TE-containing transcripts as a miRNA source

**Fig. 2:**
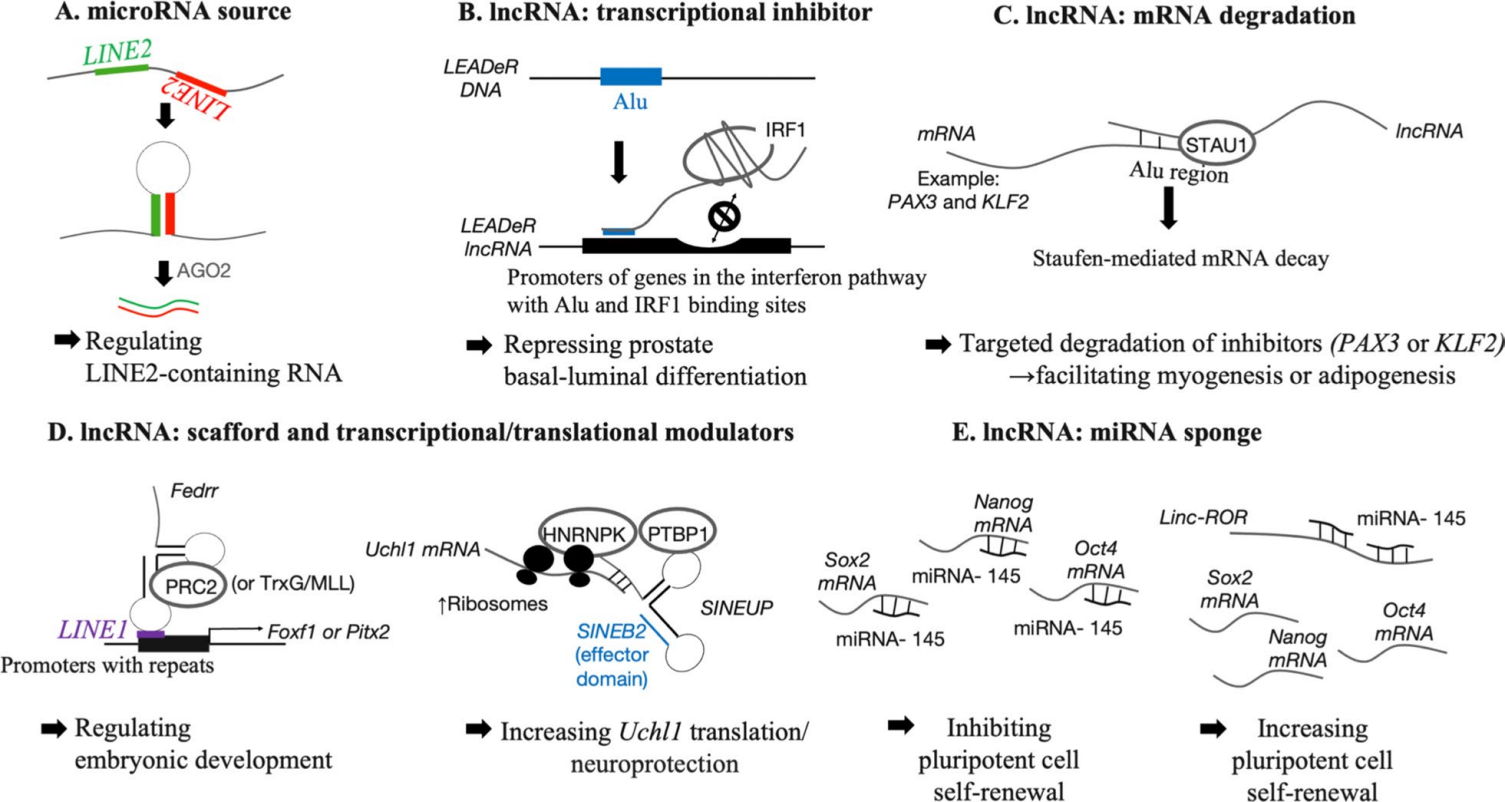
Working models of TE functions in trans. This figure summarizes the working models of TE-containing functional RNAs. When activated, they may modulate the transcriptome at the transcriptional and posttranscriptional levels. Many of these TE-derived/containing RNA-targeted genes are cell fate regulators and play important roles in development. **A** *LINE2* and inverted complementary *LINE2* sequences in the same transcript facilitate the formation of a stable double-stranded RNA structure that serves as premiRNA for generating mature, LINE2-derived microRNAs to regulate LINE2-containing transcripts. **B** An Alu-containing lncRNA *LEADeR* is recruited to promoters containing both the Alu sequence and IRF1 binding site. *LEADeR* interacts with IRF1 and may titrate IRF1 binding to the same promoter through some mechanism, leading to the silencing of differentiation-associated genes. **C** The interaction between lncRNAs and mRNAs via their complementary Alu sequences may recruit STAU1 to trigger Staufen-mediated mRNA decay. For example, *PAX3* and *KLF2* mRNAs*,* which are differentiation inhibitors*,* will be targeted by Alu-containing lncRNAs to derepress myogenesis and adipogenesis, respectively. **D** Left panel, A LINE1-containing lncRNA, *Fedrr,* selectively binds to either the transcriptional activating complex TrxG/MLL or repressive chromatin modulating complex PRC2, and targets repeat-containing promoters via the embedded LINE1 sequence to activate or repress embryonic development-related genes. Right panel, SINEB2-containing *SINEUP* lncRNAs also serve as scaffolds for translational initiation complexes, including HNRNPK and PTBP1, to increase translation. The SINEB1 sequence within *SINEUP* is important for increasing translational activity. **E** ERV-derived *Linc-ROR* serves as a miR-145 sponge to reduce the quantity of free miR-145 available to target mRNAs encoding the key pluripotent factors SOX2, OCT4 and NANOG to enforce the pluripotency status

TE-containing transcripts can be processed into microRNAs (miRNAs) [100–102]. Many of the TE-derived miRNA loci are located at the 3’ untranslated regions (3’UTRs) of protein-coding genes, which is also the target site for miRNA-mediated posttranscriptional regulation. For example, the expression of numerous Argonaute RISC catalytic component 2 protein (AGO2)-associated functional miRNAs and their target sites derived from LINE-2 sequences has been discovered in the brain cortex ([103] and references therein). L2b-derived miR-95 was significantly downregulated in the tumor biopsies of patients with glioblastoma, suggesting that the TE-centered network of miRNA targets might contribute to the normal functions of the brain (Fig. 2A).

#### TE-containing long noncoding RNAs (lncRNAs)

TE sequences are prevalent in lncRNAs. Approximately 80% of the lncRNAs identified in several studies contain at least one TE [104–106]. TEs embedded in lncRNAs may constitute the functional domain or otherwise regulate the expression, processing or localization of the host transcript (reviewed by Fort, 2021 [107]).

The lncRNAs that are involved in development may exploit embedded TEs to interact with the regulatory region of developmental genes for transcriptional modulation. During human prostate development, the canonical miRNA MIR205HG locus alternatively derives a lncRNA, long epithelial Alu-interacting differentiation-related RNA (*LEADeR*). The Alu sequence within *LEADeR* binds to the Alu element present in the regulatory sequences of its target gene, possibly by forming a paired RNA–DNA hybrid, which prevents interferon-regulatory factor 1 (IRF1) from interacting with a binding site proximal to the Alu element, thus leading to transcriptional repression of genes for luminal cell differentiation and subsequently sustaining basal cell identity [108] (Fig. 2B). In addition, LINE1 is embedded within fetal-lethal noncoding developmental regulatory RNA (*Fendrr*), a mouse lateral plate mesoderm-specific lncRNA, functioning as a putative DNA binding domain that binds to low-complexity repeats in the promoter region of target genes. The histone-modifying complexes polycomb repressive complex 2 (PRC2) and mixed lineage leukemia protein (MLL) associated with *Fendrr* are thus recruited to regulate the embryonic development of the heart and body wall by shaping the chromatin signatures of the genes involved [109, 110] (Fig. 2D).

Moreover, TE-containing lncRNAs control the expression of protein-coding genes through diverse posttranscriptional mechanisms. In contrast to the generation of miRNAs, TEs in some lncRNAs function as competing endogenous RNAs (ceRNAs, also called miRNA sponges) that recruit miRNAs with a complementary sequence from their recognition element in an mRNA, thereby stabilizing the target mRNA. For example, long intergenic non-protein-coding RNA, regulator of reprogramming (*Linc-RoR*), which is derived from human endogenous retrovirus H (HERV-H), is reported to protect the mRNAs of pluripotency-associated core transcription factors (octamer-binding transcription factor 4 (*OCT4*), SRY (sex determining region Y)-box 2 (*SOX2*), and *NANOG*) from miR-145-mediated degradation in self-renewing ESCs [111] (Fig. 2E). Recently, a primate-specific transcript isoform of the conserved protein coding gene cytochrome P450, family 20, subfamily A, polypeptide 1 (*CYP20A1*) was found to be untranslated and hence considered an lncRNA (*CYP20A1_Alu-LT*), harboring a stretch of Alu sequences at the 3’UTR to form a potential miRNA sponge [112]. The 9 miRNAs corresponding to *CYP20A1_Alu-LT* expression in primary human neurons are deduced to have mRNA targets involved in tissue-specific processes of blood coagulation and neuron development. Other cancer-relevant TE-lncRNAs modulate signaling pathways by competing for the miRNAs that target mRNAs encoding the related proteins; for example, the lncRNA hepatocellular carcinoma up-regulated long non-coding RNA (*HULC*, consisting of LTR-mammalian LTR transposon 1 A (MLT1A)) is expressed at high levels in liver cancer, and BRAF-activated nonprotein coding RNA (*BANCR*, consisting of LTR-MER41B) has been shown to act as a sponge for miR-372 to derepress protein kinase cAMP-activated catalytic subunit beta (PRKACB) and for miR-338-3p to derepress insulin-like growth factor 1 receptor (IGF1R) in esophageal squamous cell carcinoma [113–115].

*SINEUP,* a type of lncRNA containing SINE elements that upregulate the translation of target mRNAs, is a bipartite antisense RNA with one effector domain containing the SINE element and an RNA-binding domain to recognize the target mRNA by complementary pairing with the 5’ end sequence surrounding the AUG start codon. The underlying mechanism was recently suggested: the SINE element domain contributes to the recruitment of the RNA binding proteins polypyrimidine tract-binding protein 1 (PTBP1) and heterogeneous nuclear ribonucleoprotein K (HNRNPK), leading to the translocation of the paired lncRNA and target mRNA into the cytoplasm to facilitate the assembly of translational initiation complexes [116]. A lncRNA antisense to mouse ubiquitin carboxy-terminal hydrolase L1 (*Uchl1*) containing the *SINEUP* feature has been identified. It increases the translation of Uchl1/Park5, which is essential for brain function and particularly for neuron maintenance [117] (Fig. 2D, right panel). Moreover, a human *SINEUP* lncRNA discovered in the brain transcriptome was shown to upregulate the translation of protein phosphatase 1 regulatory subunit 12A (PPP1R12A), a downstream effector of inhibitory glutamate receptor delta-1 (GluD1), in postsynaptic cortical pyramidal neurons [118]. This unique regulatory function of TE-containing lncRNAs has recently prompted scientists to design and apply synthetic *SINEUP* to increase the translation of proteins of interest [119].

One type of TE-containing lncRNA is involved in mRNA degradation, specifically through the Staufen-mediated mRNA decay (SMD) mechanism [120, 121]. STAU (Staufen protein) binds to double-stranded RNA that is formed by imperfect base pairing between an Alu element in the 3’UTR of the target mRNA and another Alu element within a lncRNA (named half-STAU1-binding site RNA, *½-sbsRNA*) to elicit the SMD mechanism by recruiting up-frameshift suppressor 1 (UPF1) and UPF2, the core factors in the mRNA degradation pathway. Examples of development-relevant *½-sbsRNA* lncRNAs in humans include lncRNAs that target the mRNA of *PAX3* (encoding the myogenesis inhibitor paired box gene 3), which is implicated in myogenesis [122], lncRNAs that target the Krüppel-like factor 2 (*KLF2*) mRNA (the KLF2 protein, in turn, negatively regulates the adipogenic gene *PPARγ*) in adipogenesis [123], and lncRNAs involved in mouse myogenesis [124], with *SINE B1*, *B2*, and *B4* subfamilies (except for the primate-specific Alu) among the putative mouse *½-sbsRNAs* targeting the 3’UTRs of several mRNAs for degradation (Fig. 2C).

### The gray zone between *cis *and *trans:* native elongating TE-containing functional RNAs modulate X chromosome inactivation and reactivation

*Xist* is a well-known lncRNA that is critical for initiating and was recently shown to also be important for maintaining X chromosome inactivation (Xi) in female cells. It consists of various tandem repeats (A–F), possibly originating from a variety of TE families, including ERVs, LINEs and SINEs. When *Xist* “coats” the inactivating X chromosome, these TE components within the RNA sequence are required for the recruitment of several transcriptional silencers, polycomb repressive histone modifiers, and other factors related to the establishment and maintenance of X chromosome inactivation ([125]; reviewed by Pintacuda, 2017 [126]).

Additionally, relevant to the X chromosome dosage compensation process but having the opposite function, primate-specific Xact competes with Xist and enables erosion of the Xi chromosome, which results in X chromosome reactivation (XCR) [112–114]. When we studied Xact lincRNA sequences in the UCSC genome browser on Human Feb. 2009 (GRCh37/hg19) Assembly, we identified its embedded TE elements, including LTR9B, AluY, and MLT1J (unpublished observation). Despite the presence of TEs in *Xact*, the function and interacting proteins of TEs have yet to be determined. The ERV1 LTR9B-derived sequence binds to OCT4 and SOX2 proteins, which subsequently modulate the expression of various pluripotent genes [127]. Further investigations of whether *LTR9B-*containing *Xact* is also involved in the initiation or maintenance of pluripotency, in addition to its X chromosome reactivation function, would be interesting.

### TE-containing *cis-*regulatory elements

Apart from being incorporated into functional RNAs to execute trans-acting functions, TE DNA sequences are also substantially involved in modulating gene expression by serving as binding sites for heavily weighted transcription factors, epigenetic modifiers or insulator binding proteins. For example, thousands of ERVs carry functional tumor suppressor protein P53 binding sites and regulate nearby genes, especially in the event of DNA damage [128, 129]. The expansion of these mobile carriers of transcriptional modulators and chromatin looping factors also provided opportunities for strain-specific or species-specific transcriptional networks and phenotypes [130].

#### TE-containing enhancers

**Fig. 3:**
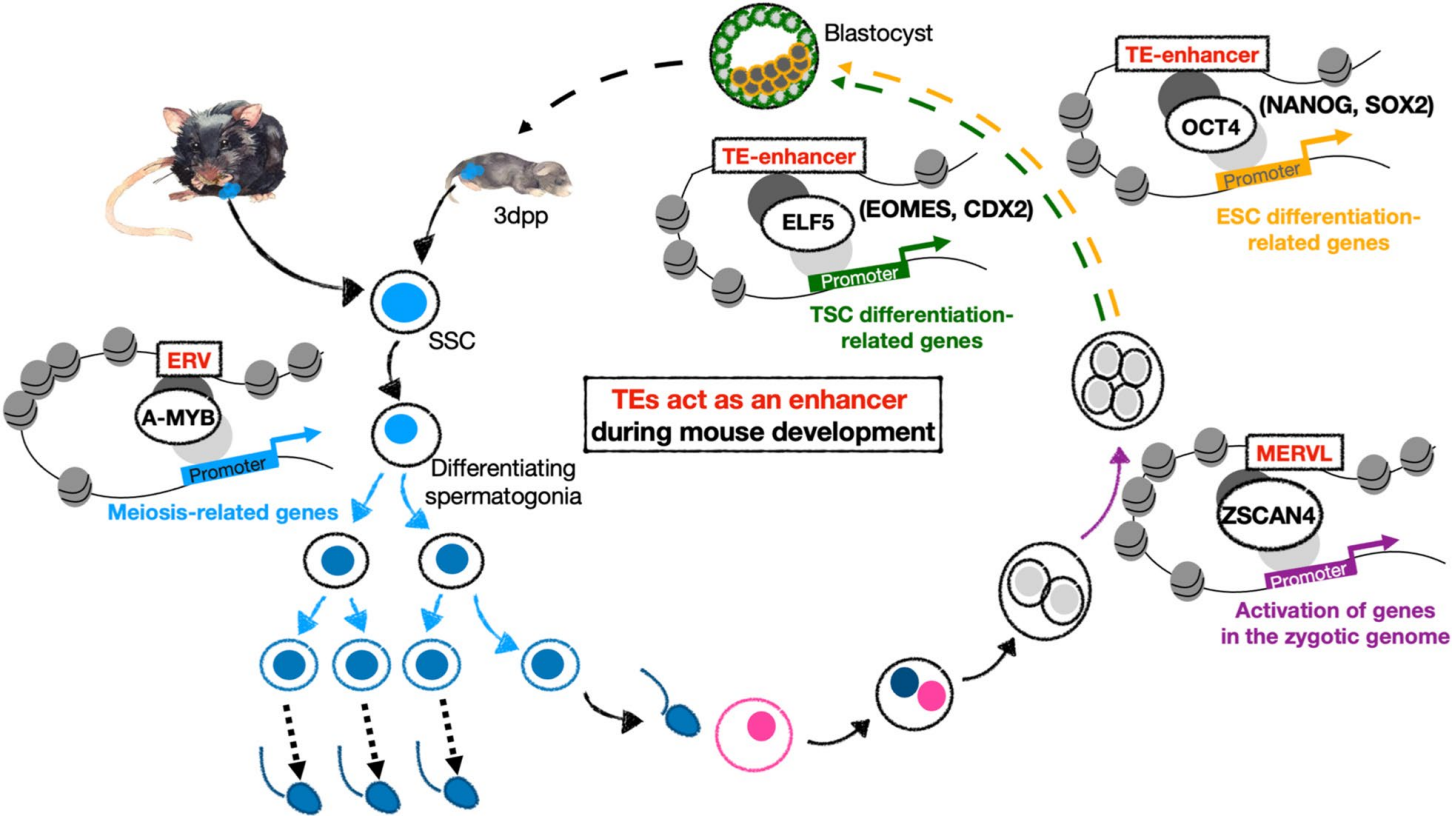
Some TEs function as enhancers during mouse development. TEs play a role in determining cell fate during different phases of mouse development by regulating transcription through *cis* activation. In two-cell to four-cell preimplantation embryos, ZSCAN4 binds to MERVLs to initiate zygotic genome activation. When the embryo develops from the four-cell stage into a blastocyst, TE-containing enhancers recruit different transcription factors to differentiate the embryo into ESCs and TSCs. During ESC differentiation, OCT4, NANOG and SOX2 are recruited by TE-containing enhancers. During TSC differentiation, ELF5, EOMES and CDX2 are recruited by other TE-containing enhancers. During the mitosis-to-meiosis transition, spermatogonia differentiate into the stages within PSs and round spermatids, and enhancer-like ERVs recruit A-MYB to facilitate meiosis-related gene expression. TEs, transposable elements; ZSCAN4, zinc finger and SCAN domain containing protein 4C; MERVL, mouse endogenous retrovirus L; ESC, embryonic stem cell; OCT4, octamer-binding transcription factor 4; SOX2, SRY (sex determining region Y)-box 2; TSC, trophoblast stem cell; ELF5, E74-like factor 5 (ETA domain transcription factor); EOMES, eomesodermin; CDX2, caudal-type homeobox transcription factor 2; dpp, days postpartum; SSC, spermatogonial stem cell; A-MYB, A-myoblastosis protein

After fertilization, different ERVs are activated at different stages of preimplantation embryo development [131]. For instance, oocytes/zygotes express the ERVK family member RLTR40, while zygotes/2-cell stage embryos express the ERVL-MaLR family member MTA. When 2-cell embryos differentiate to the 4-cell stage, the embryo faces zygotic genome activation (ZGA), the stage in which the embryos produce necessary RNA and protein from their own genome and gradually wean from those inherited from the oocytes. The expression and enhancer activities of MERVL, as documented by increased chromatin accessibility, are both critical for ZGA. Embryonic development is arrested upon MERVL deficiency [132]. The expression of 3’ downstream proximal genes, including many cleavage-stage specific genes (cleavage genes), is modulated by a mechanism depending on MERVL accessibility. [133]. Upon activation by DUX4 and Zscan4c, key factors in the ZGA stage, MERVL plays an important role in cleavage gene regulation [131] (Fig. 3). Moreover, MERVL is also involved in translational modulation at the ZGA stage [134]. In summary, MERVL is an ERV that is specifically expressed and serves as an active enhancer during ZGA to modulate cleavage genes crucial for zygote development [132].

Under in vitro culture conditions, ESCs and trophoblast stem cells (TSCs) can be derived from blastocyst-stage embryos. ESCs and TSCs are responsible for the development of fetal and extraembryonic tissue, respectively. Researchers performed assay for transposase-accessible chromatin using sequencing (ATAC-seq), ChIP-seq and promoter capture Hi-C (PCHi-C) to study ESCs and TSCs and suggested that they contained distinct TE subfamilies that functioned as enhancers to regulate gene expression and determine cell differentiation. By performing ATAC-seq and H3K27ac enrichment analyses and confirming binding by at least one of the three key transcription factors (NANOG, OCT4 and CTCF in ESCs; ELF5, EOMES and CDX2 in TSCs), a subset of TEs were defined as “ESC/TSC TE enhancers” [98, 99] (Fig. 3). The activity of these TE enhancers is more restricted in ESCs/TSCs than that of non-TE enhancers. Using PCHi-C to analyze the correlation between enhancers and gene expression, researchers showed that TE enhancer-interacting genes displayed higher expression levels in both ESCs and TSCs than genes interacting with non-TE enhancers [98]. Furthermore, the analysis of gene expression levels across a wide array of tissues indicated that genes interacting with TE enhancers were almost exclusively expressed in ESCs or TSCs [135]. Genetic and chromatin analyses suggested that TE enhancers may be used to support lineage-specific expression of a subset of genes in early embryonic development [136]. Thus, TE enhancers play a critical role in early embryonic development and differentiation.

In male germ cells, dramatic reorganization of epigenomic modifications occurs during the transition from mitosis to meiosis in spermatogenesis. Enhancer-like ERVs such as RLTR10 also recruit A-myoblastosis protein (A-MYB) to facilitate germ cell differentiation (Fig. 3). In vitro dual-luciferase assays indicated that A-MYB dramatically increased the enhancer activity of ERVs. Interestingly, A-MYB depletion led to a decrease in the H3K27ac level, suggesting that A-MYB plays a role in the activation of enhancer-like ERVs. Human ERVs also exhibit this enhancer-like function in spermatogenesis. MER57E3 (ERV1) and LTR5B (ERVK) are enriched with H3K27ac in pachytene spermatocytes (PSs). Moreover, ERV1 and ERVK also contain binding motifs for A-MYB, which is expressed at high levels in human spermatocytes, suggesting that enhancer-like ERVs have similar activation mechanisms in human spermatogenesis. A small fraction of super-enhancers required for the mitosis-to-meiosis transition are also ERV-containing, A-MYB-binding enhancers, associated with the activation of meiosis-associated genes [137, 138] (personal communication between Prof. Satoshi Namekawa and Prof. Shau-Ping Lin).

Enhancer-like ERVs are also associated with the diversity of gene expression in different species during evolution. Forty-eight mouse-specific genes were identified among 381 enhancer-like ERV-adjacent genes. Moreover, by analyzing rodent-specific ERVKs, researchers found that enhancer-like ERVs show differences in both copy numbers and genome distributions between mice and rats. In humans, 52 of 66 enhancer-like MER57E3 sequences are located at the first intron of a zinc finger protein. Among them, 47 of 52 were KRAB-ZFPs, suggesting a coevolutionary mechanism. This phenomenon was also observed in neuronal differentiation-briefly, KRAB-ZFPs partner with ERVs, regulating gene expression in human neurons [11]. The levels of enhancer-like MER57E3 and ERVK-adjacent genes are upregulated during the mitosis-to-miosis transition in humans. Similar to those in mice, 61 of 138 enhancer-like ERV-adjacent genes were identified as primate-specific genes. Thus, ERVs have rapidly evolved in mammals to regulate several function-specific genes in the host genome [137, 138].

In the immune system, Chuong et al*.* showed that ERVs are significantly enriched in numerous interferon (IFN) regulatory elements in different mammalian genomes [139]. As a result, ERVs are considered IFN-inducible enhancers, and ERVs are strongly correlated with the innate immunity-associated IFN response [139]. Additionally, TEs are more highly enriched near immune genes in cytotoxic T cells and CD8^+^ cells than in nonimmune cells, supporting the hypothesis that the immune response may depend on the function of TEs as enhancers to rapidly activate immune pathways. In adaptive immune cells, TEs have been reported to function as enhancers that regulate putative CD8^+^ T cell immunity [140]. Ye et al. [140] employed genome-wide chromatin analysis and ATAC-seq to assess the contribution of TEs to T lymphocyte development. Researchers divided the T cell enhancer region into three distinct domains, an accessible core, proximal flanking region, and distal flanking region, to further elucidate the regulatory functions of different TEs. The authors proposed that different TEs may be predisposed to contribute distinct regulatory functions. For example, ERVs enriched at enhancer cores may provide transcription factor binding sites, while B1 SINEs enriched in enhancer flanks are more likely to facilitate chromatin organization. Moreover, SINEs are associated with high levels of the histone mark H3K4me1 [140], which is thought to serve as an enhancer [141]. Ye et al. [140] further suggested that epigenetic dysregulation of TE-derived enhancers may result in inappropriate activation of CD8^+^ T cells, which further shows a high correlation with TEs, especially those in the ERV subfamily [140].

#### TEs function as insulators and modulate the 3D chromatin conformation

**Fig. 4:**
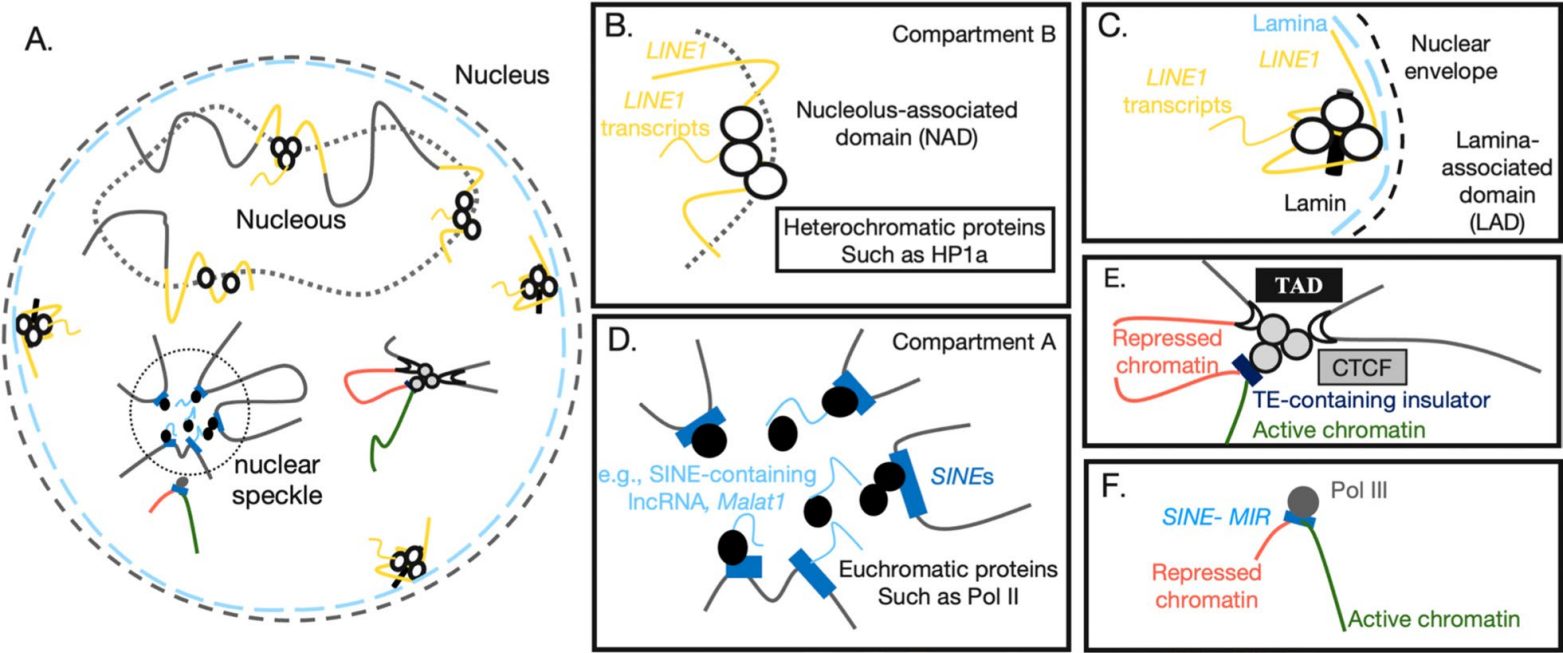
TEs function as insulators and modulators of the 3D chromatin conformation. **A** TE sequences facilitate the establishment of 3D chromatin structures in the nucleus, including the formation of nuclear speckles and nucleoli. **B** LINE1 serves as a seed to recruit heterochromatic proteins such as HP1a. HP1a and/or LINE1 transcripts may also attract each other and undergo a phase-separation mechanism from LINE1-enriched compartment B in the nucleolus. **C** Lamin also targets LINE1 sequences to form heterochromatin by the inner nuclear membrane. **D** SINEs serve as seeds to recruit euchromatic proteins such as Pol II to promote transcription. Pol II and/or SINE B1 transcripts may further attract each other and undergo a phase-separation mechanism from nuclear speckles (SINE-enriched nuclear compartment A), activating gene expression. **E** TEs also serve as insulators and attract some proteins, such as CTCF or Pol III, to block enhancer accessibility and insulate gene expression. **F** Mammalian-wide interspersed repeats (MIRs), which belong to the SINE family, can be targeted by Pol III to function as insulators

Apart from attracting epigenetic modifiers to spread histone modifications and sometimes DNA methylation and therefore affecting the transcriptional activities of their neighboring genes, the involvement of TE sequences as insulators or factors contributing to the modulation of 3D chromatin architecture has been revealed over the last 2 decades (reviewed by Nishihara, 2019 [142]) (Fig. 4A–F). For example, the on-site transcripts of retrotransposon SINE B2 repeats function as insulators to prevent enhancer access and thus provide domain boundaries during organogenesis [143]. The 11-zinc-finger CCCTC-binding factor (CTCF) is a well-known *trans*-acting transcriptional repressor and a critical mediator of chromatin looping. Some retrotransposons contain CTCF binding sites, and with their expansion in a particular host genome, the 3D chromatin looping structures change with them, sometimes generating species-specific chromatin looping structures [144] (Fig. 4E). In addition to CTCF-dependent modulation of the 3D genome architecture, a recent study also identified another transposable element, mammalian-wide interspersed repeats (MIRs), serving as insulator elements in immune cells via a CTCF-independent pathway [145] (Fig. 4F). Homotypic clustering of L1 and B1/Alu transcripts reorganizes the 3D genome into higher-order compartments [146] (Fig. 4B–D). From the perspective of evolution, evolutionarily young TE subfamilies such as L1PA and AluY are significantly enriched at topological associating domain (TAD) boundaries in the developing cortex of human brains, while older TE subfamilies such as MIR, LINE-2, Charlie, and MaLR are enriched at TAD boundaries conserved across species [130].

## Conclusions

TEs can cause genomic instability via transposition dependent and independent mechanisms, potentially resulting in cell death or cancer formation. DNA methylation, histone modifications and functional RNA machineries are evolved to modulate TE at the cell type dependent and developmental stage-dependent manner. Cumulative evidence also suggested physiological significance of TE sequence-dependent mechanisms that provide novel layers of transcriptome modulation in epigenetic, nuclear architecture and post-transcriptional levels. These include TE-containing transcripts serving as miRNA sources, miRNA sponges and functional RNAs for guiding DNA binding proteins; TE-containing cis regulatory element as enhancers, promoters and insulator to regulate gene expression. In addition, TEs also act as genetic accelerators of evolution, contributing to the genome size, species-specific gene regulatory network rewiring, morphological innovation. Further understanding of TE related physiological functions and pathological etiology could lead to novel therapeutic opportunities.

## Data Availability

Not applicable.

## Abbreviations

AGO2: Argonaute RISC catalytic component 2 protein
APC: Adenomatous polyposis coli protein
ARC: Activity-regulated cytoskeleton-associated protein
A-MYB: A-myoblastosis protein
ATAC-seq: Assay for transposase-accessible chromatin using sequencing
*BANCR*: BRAF-activated nonprotein coding RNA
BRCA2: Breast cancer type 2 susceptibility protein
CDX2: Caudal-type homeobox transcription factor 2
CD8: Cluster of differentiation 8
ceRNA: Competing endogenous RNAs
ChIP: Chromatin immunoprecipitation
CTCF: The 11-zinc-finger CCCTC-binding factor
*CYP20A1*: Cytochrome P450, family 20, subfamily A, polypeptide 1
DNMTs: DNA methyltransferases
DNMT3L: DNA methyltransferases 3 like
DSBs: Double-strand breaks
DUX4: Double homeobox protein 4
ELF5: E74-like factor 5 (ETA domain transcription factor)
EOMES: Eomesodermin
ERVs: Endogenous retrovirus
ESCs: Embryonic stem cells
*Fendrr*: Fetal-lethal noncoding developmental regulatory RNA
GluD1: Glutamate receptor delta-1
HERV-H: Human endogenous retrovirus H
HNRNPK: Heterogeneous nuclear ribonucleoprotein K
*HULC*: Hepatocellular carcinoma up-regulated long non-coding RNA
H3K4me1: Histone H3 lysine 4 monomethylation
H3K4me3: Trimethylation of lysine 4 on histone H3 protein subunit
H3K9me2/3: Dimethylation/trimethylation of lysine 9 on histone H3 protein subunit
H3K27ac: Histone H3 lysine 27 acetylation
H3K27me3: Histone H3 lysine 27 trimethylation
IAPs: Intracisternal A-type particles
IGF1R: Insulin-like growth factor 1 receptor
IFN: Interferon
IRF1: Interferon-regulatory factor 1
KAP1: KRAB-associated protein-1 (also known as TRIM28)
*KLF2*: Krüppel-like factor 2
KRAB-ZFPs: Krüppel-associated box zinc finger proteins
KRAB-ZNF-KAP1: KRAB-zinc finger and KAP1 protein complex
*LEADeR*: Long epithelial Alu-interacting differentiation-related RNA
*Linc-RoR*: Long intergenic non-protein-coding RNA, regulator of reprogramming
LINEs: Long interspersed nuclear elements
lncRNAs: Long noncoding RNAs
LTRs: Long terminal repeats
L2b: LINE-2 b
MEL: Murine erythroleukemia cells
MERV: Murine endogenous retrovirus
miR: MicroRNA
miRNAs: MicroRNAs
MIRs: Mammalian-wide interspersed repeats
MIR205HG: MIR205 host gene microRNA
MLL: Mixed lineage leukemia protein
MLT1A/MLT1J: Mammalian LTR transposon 1 A/J
mRNA: Messenger RNA
MuERV-L: Murine endogenous retrovirus-leukemia protein
OCT4: Octamer-binding transcription factor 4
*PAX3*: Paired box gene 3
PCHi-C: Promoter capture Hi-C
piRNA: PIWI-interacting RNA
PIWI: P-element-induced wimpy testis protein
PNMA: Paraneoplastic Ma antigens protein
*PPARγ*: Peroxisome proliferator-activated receptor gamma
PPP1R12A: Protein phosphatase 1 regulatory subunit 12A
PRC2: Polycomb repressive complex 2
PRKACB: Protein kinase cAMP-activated catalytic subunit beta
PSs: Pachytene spermatocytes
PTBP1: Polypyrimidine tract-binding protein 1
RAG1: Recombination activating 1 protein
RB1: Retinoblastoma protein 1
RNAi: RNA interference
RSSs: Recombination signal sequences
SAM: S-adenyl methionine
SASPase/ASPRV1: Skin-specific retroviral-like aspartic protease/aspartic peptidase retroviral like 1 protein
SCAN: SRE-ZBP, CTfin-51, AW-1, 2 Number 18 cDNA protein
SEs: Superenhancers
SETDB1: SET domain bifurcated histone lysine methyltransferase 1
SINEs: Short interspersed nuclear elements
SMD: Staufen-mediated mRNA decay
SOX2: SRY (sex determining region Y)-box 2
TAD: Topological associating domain
TEs: Transposable elements
TRIM28: Tripartite motif-containing 28 protein
TSCs: Trophoblast stem cells
Uchl1/Park5: Ubiquitin carboxy-terminal hydrolase L1
UHRF1: Ubiquitin-like, containing PHD and RING finger domains protein 1
UPF1/2: Up-frameshift suppressor 1/2
XCR: X chromosome reactivation
Xi: X chromosome inactivation
ZGA: Zygotic genome activation
Zscan4c: Zinc finger and SCAN domain containing protein 4C
*½-sbsRNA*: Half-STAU1-binding site RNA
3’UTR: 3′ Untranslated region
5mC: 5-Methylcytosine
